# RT-AFNet: A Hybrid ResNet-Transformer Architecture with Multi-Scale Fusion for Atrial Fibrillation Detection

**DOI:** 10.3390/bios16050275

**Published:** 2026-05-09

**Authors:** Xinyu Hu, Qingqing Duan, Yuwei Zhang, Caiyun Ma, Chang Yan, Chengyu Liu

**Affiliations:** 1 School of Computer Science, Jiangsu University of Science and Technology, Zhenjiang 212003, China; 232210404103@stu.just.edu.cn (X.H.); 232210404101@stu.just.edu.cn (Q.D.); 2 The State Key Laboratory of Bioelectronics, School of Instrument Science and Engineering, Southeast University, Nanjing 210096, China; 101300447@seu.edu.cn (C.M.); chengyu@seu.edu.cn (C.L.); 3 School of Future Technology, Shandong University, Jinan 250061, China; cyan@sdu.edu.cn

**Keywords:** atrial fibrillation (AF), electrocardiogram (ECG), Residual Neural Network (ResNet), transformer

## Abstract

Atrial fibrillation (AF) is a prevalent cardiac arrhythmia associated with an elevated risk of severe complications, including stroke and heart failure. Due to its paroxysmal nature and the inherent complexity of electrocardiogram (ECG) signals, developing highly accurate and robust automated detection methods remains a critical challenge. To address the limitations of existing models in simultaneously capturing local morphological anomalies and long-range temporal dependencies, we proposed RT-AFNet, a novel hybrid ResNet-Transformer architecture. Specifically, RT-AFNet integrated the robust local feature extraction capabilities of a Residual Neural Network (ResNet) backbone with the global temporal modeling power of a lightweight self-attention mechanism. Furthermore, a multi-scale feature fusion strategy was introduced to optimize feature representation. The proposed RT-AFNet model was evaluated on three public AF databases: the China Physiological Signal Challenge 2018 (CPSC2018), the PhysioNet/Computing in Cardiology Challenge 2017 (CinC2017), and the MIT-BIH Atrial Fibrillation Database (MIT-BIH AF). The proposed model achieved F1 scores of 99.76%, 97.47%, and 96.20%, along with area under the curve (AUC) values of 99.97%, 98.98%, and 98.28% on the three datasets, respectively. These results demonstrate that the proposed architecture exhibits excellent generalization ability and stability across different databases, providing a robust and reliable deep learning solution for automated AF screening.

## 1. Introduction

Atrial fibrillation (AF) is the most prevalent sustained arrhythmia in clinical practice, characterized by rapid and irregular atrial contractions [1]. Globally, over 37 million adults are affected by AF, with a continuously rising prevalence driven by population aging [2]. This condition significantly increases the risk of cardioembolic stroke, and is recognized as a leading preventable cause of this life-threatening complication [3,4]. The clinical presentation of AF is highly heterogeneous. Paroxysmal AF, in particular, manifests as short, unpredictable episodes with subtle or even asymptomatic manifestations in the early stage, making it easily missed during standard short-duration electrocardiogram (ECG) examinations. Therefore, long-term continuous cardiac monitoring is critical for accurate and timely AF diagnosis [5].

**Figure 1:**
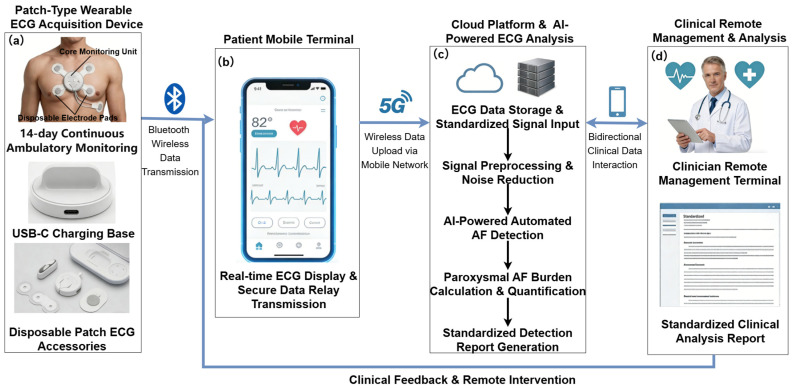
Schematic of the patch-type wearable ECG-based intelligent AF monitoring system. Core modules: (**a**) 14-day continuous ambulatory ECG acquisition device; (**b**) patient mobile terminal for ECG monitoring and data transmission; (**c**) cloud platform for AI-powered AF detection; (**d**) clinician terminal for remote patient management.

Traditional Holter monitors, the gold standard for long-term ECG recording, are limited to a 24–48 h recording window, which often fails to capture brief paroxysmal AF episodes. These devices are also bulky and uncomfortable for patients, interfering with daily activities. In recent years, patch-type and wearable ECG devices have emerged as promising tools for AF screening. Their compact, lightweight design enables continuous monitoring for up to 14 days, making them well-suited for community-based screening and long-term home monitoring [6,7]. These devices are typically integrated into a cloud-based intelligent AF monitoring system, whose general workflow is illustrated in Figure 1. Such systems realize end-to-end closed-loop management from long-term ECG signal acquisition, AI-powered automated AF detection, to remote clinical intervention, providing strong technical support for early AF screening and diagnosis. In addition to continuous long-term monitoring devices, another important class of ECG monitors designed for intermittent but repeated short-term measurements (e.g., approximately 1 min recordings, once or twice daily), akin to home blood pressure monitoring, has emerged. These devices have proven particularly effective in detecting brief and isolated paroxysmal AF episodes [8]. By integrating into a patient’s daily routine, they offer a low-burden alternative for long-term surveillance and can also be incorporated into the intelligent monitoring paradigm.

However, the massive volume and inherent complexity of data from both continuous and intermittent wearable devices pose significant challenges for automated AF detection algorithms. The inherent signal-level constraints of wearable ECG acquisition further compound these challenges. Dynamic recordings obtained during free-living conditions are inevitably corrupted by motion artifacts, myoelectric noise, and baseline wander, which can obscure or mimic the subtle electrocardiographic signatures that are critical for reliable AF diagnosis, particularly the disappearance of P waves [9]. Consequently, denoising algorithms designed for wearable dynamic ECG have become an essential prerequisite for accurate rhythm analysis. In parallel, the limited computational resources and strict power budgets of battery-operated wearable platforms impose uncompromising demands on model efficiency. Practical algorithms must operate within tight memory footprints and energy envelopes, making the compression of deep learning architectures a pivotal consideration for on-device deployment [10]. Collectively, these observations highlight that an effective wearable AF screening algorithm must not only be accurate and generalizable, but also inherently robust to real-world noise and computationally lightweight.

Generally, there are two main approaches for AF detection: traditional machine learning methods and deep learning-based methods.

Traditional machine learning methods often rely on hand-crafted features extracted from ECG signals. For instance, Datta et al. [11] developed a multi-layer binary classifier using hand-crafted statistical, morphological, and heart rate variability features from single-lead ECG signals. However, manual feature extraction is time-consuming, has limited representation capacity for complex data, and performs poorly in noisy and high-variability scenarios. Similarly, Athif et al. [12] proposed a rule-based algorithm using statistical and morphological features, yet it only achieved a sensitivity of 77.5% and demonstrated poor adaptability to low-quality single-lead signals.

In contrast, deep learning-based methods aim to automatically learn features and capture temporal dependencies. Kiranyaz et al. [13] introduced a 1D convolutional neural network (1D-CNN) model for patient-specific ECG classification, which effectively captured local spatiotemporal features; however, it failed to learn long-range temporal dependencies associated with AF. To address sequence modeling, Schuster et al. [14] proposed a bidirectional recurrent neural network (RNN) structure to process sequence information. Nevertheless, this approach suffered from vanishing gradient problems as sequence length increases, making it ineffective for long-duration AF signals. Shewalkar et al. [15] compared the performance of long short-term memory (LSTM) and gated recurrent unit (GRU) networks for AF detection. Although these models captured partial temporal dependencies, their accuracy degraded significantly when modeling ultra-long ECG signals. More recently, the Transformer architecture proposed by Vaswani et al. [16] used global self-attention to capture intra-sequence relationships. Despite its effectiveness, a pure Transformer model had high computational complexity, which hindered its deployment on resource-constrained wearable devices.

To address the aforementioned limitations, this study proposed a RT-AFNet architecture for automatic AF detection from ECG signals. This architecture effectively integrated the strengths of Residual Neural Network (ResNet) in local feature extraction and the Transformer self-attention mechanism in long-range dependency modeling. By embedding lightweight self-attention modules into the key layers of the ResNet backbone, it simultaneously captured the local morphological features and global temporal context of ECG signals. To verify the robustness and generalization ability of the model, we conducted experiments on three public AF datasets: China Physiological Signal Challenge 2018 (CPSC2018), PhysioNet/Computing in Cardiology Challenge 2017 (CinC2017), and MIT-BIH Atrial Fibrillation Database (MIT-BIH AF). This research aims to provide a high-accuracy, efficient AF detection solution for wearable ECG devices, supporting the development of early AF screening and long-term monitoring technologies.

The main contributions of this paper are summarized as follows:

1. **Spatiotemporal Joint Representation Learning Framework.** We designed a RT-AFNet architecture, which combines the local feature extraction power of a ResNet backbone with the global temporal modeling capability of a lightweight self-attention mechanism. By integrating attention modules in key layers (specifically, Layer 2 and Layer 3) to capture long-range dependencies, which refer to temporal relationships spanning multiple heartbeats (i.e., minute-scale temporal dynamics), and implementing hierarchical feature fusion, we built an end-to-end unified learning framework for ECG-based AF detection.
2. **Multi-Scale Feature Fusion and Adaptive Robust Training Strategy**. We introduced a comprehensive multi-scale feature fusion mechanism alongside an adaptive training strategy to enhance the model’s representation capacity and optimize learning dynamics. The fusion module dynamically aggregates global pooling representations, multi-resolution temporal semantics, attention-weighted vectors, and statistical features to accurately map irregular rhythm patterns. Weighted Cross-Entropy (WCE) Loss was used to address class imbalance, a layer-wise learning rate schedule is implemented for different modules, and data augmentation techniques including Gaussian noise addition and random masking were applied to achieve balanced improvement in precision and recall.
3. **Validation of generalization ability based on multi-source datasets.** We conducted comprehensive empirical validations of the proposed framework across three diverse public AF datasets. This demonstrates the architecture’s capacity to maintain stable, high-accuracy performance across varying signal qualities and diverse patient populations, thereby verifying its reliability for a wide range of real-world clinical application scenarios.

The remainder of this paper is organized as follows. Section 2 describes the materials and methods, including the three public AF databases, the preprocessing workflow, and the proposed RT-AFNet architecture. Section 3 presents the experimental results, covering classification performance, ablation analysis, cross-database generalization, error analysis, and complexity evaluation. Section 4 discusses the findings, compares RT-AFNet with state-of-the-art methods, and analyzes its behavior on confounding arrhythmias. Section 5 concludes the paper and outlines directions for future work.

## 2. Materials and Methods

### 2.1. Databases

CPSC2018 Database: The China Physiological Signal Challenge (CPSC) 2018 database was released during the 7th International Conference on Biomedical Engineering and Biotechnology [17]. This large-scale, multi-label 12-lead ECG database was collected collaboratively from 11 hospitals in China. It contains 6877 12-lead ECG recordings, each with a duration of 6 s to 60 s and a sampling rate of 500 Hz. All recordings are annotated with one of nine diagnostic labels: normal sinus rhythm (NSR), AF, first-degree atrioventricular block (I-AVB), left bundle branch block (LBBB), right bundle branch block (RBBB), premature atrial contraction (PAC), premature ventricular contraction (PVC), ST-segment depression (STD), and ST-segment elevation (STE).

CinC2017 Database: The CinC2017 database is a public dataset released for the PhysioNet/Computing in Cardiology Challenge 2017 [18], designed to advance the development of automatic AF detection algorithms for short single-lead ECG recordings. The database contains 8528 single-lead ECG segments, each sampled at 300 Hz with a duration of 9 s to 60 s. The recordings were acquired using an AliveCor Kardia device (AliveCor, Inc., Mountain View, CA, USA), which generates a single-lead tracing morphologically similar to standard Lead I. Each recording is annotated into one of four categories: NSR, AF, other rhythms, and noisy signals. For the CPSC2018 and CinC2017 datasets, we only included recordings labeled as NSR or AF due to the limited number of samples from other rhythm types.

**Figure 2:**
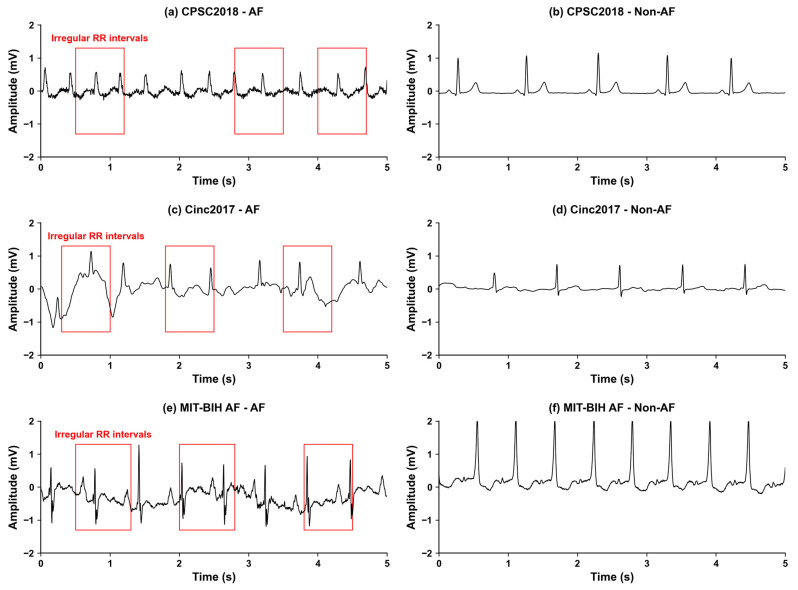
Example ECG waveforms of AF and Non-AF sinus rhythm from three public datasets. (**a**) AF rhythm sample from the CPSC2018 dataset; (**b**) Non-AF normal sinus rhythm sample from the CPSC2018 dataset; (**c**) AF rhythm sample from the CinC2017 dataset; (**d**) Non-AF normal sinus rhythm sample from the CinC2017 dataset; (**e**) AF rhythm sample from the MIT-BIH AF dataset; (**f**) Non-AF normal sinus rhythm sample from the MIT-BIH AF dataset. The vertical axis represents the ECG signal amplitude (mV), and the horizontal axis represents time (s).

MIT-BIH AF Database: The MIT-BIH Atrial Fibrillation Database is a public dataset hosted on the PhysioNet platform, specifically designed for the development and validation of AF detection algorithms [19,20]. It contains 23 long-term ECG recordings (21 from patients with paroxysmal AF), with a sampling frequency of 250 Hz and an average duration of 10 h 15 min per recording. The annotations include four rhythm types: AF, normal sinus rhythm (N), AV junctional rhythm (J), and atrial flutter (AFL). In this study, we combined AFL samples into the AF category and classified N and J rhythms into the Non-AF group. Example ECG visualizations from each dataset are shown in Figure 2.

### 2.2. Preprocessing

To ensure consistency across all three datasets, we applied a unified preprocessing pipeline. Single-lead analysis was adopted because the primary target of this study is wearable and patch-based ECG devices for long-term AF screening, the vast majority of which record only a single-lead signal. Among all single leads, Lead II was chosen for the CPSC2018 and MIT-BIH AF datasets because it provides the clearest visualization of atrial activity (P waves) and ventricular depolarization (QRS complexes), making it the preferred rhythm monitoring lead in clinical practice. For the CinC2017 dataset, the original single-lead recordings, which are morphologically similar to standard Lead I, were used directly. All signals were resampled to 400 Hz and filtered using a fourth-order zero-phase Butterworth band-pass filter with a passband of 0.5–45 Hz to remove baseline wander and high-frequency noise. The filtered signals were then segmented into 10 s non-overlapping windows (4000 samples per window); any incomplete window shorter than 10 s was discarded. The preprocessed samples were divided into training, validation, and test sets at a ratio of 70:15:15 using stratified sampling. Given the limited number of AF samples in the datasets and to enhance model robustness, data augmentation was applied only to the AF samples in the training set. This involved employing Gaussian noise (2% of the signal’s standard deviation) and random masking (2–10% linear interpolation filling), both with an augmentation ratio of 2.0, thereby doubling the number of AF samples in the training set. Table 1 summarizes the final data volumes after preprocessing.

### 2.3. Proposed Model

**Figure 3:**
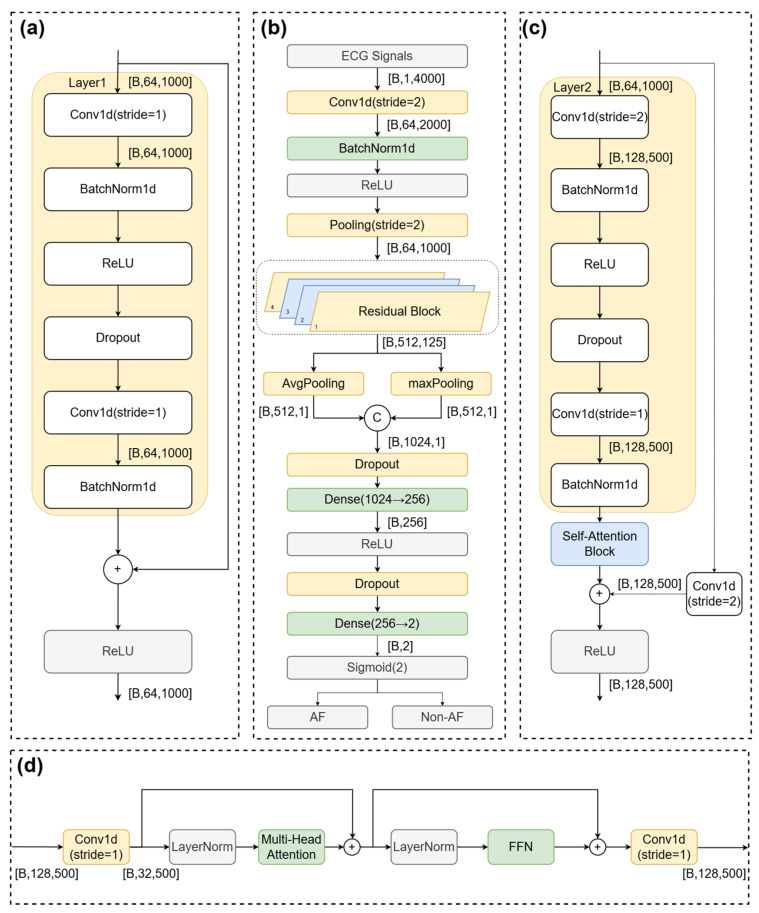
Overall architecture of the proposed RT-AFNet model for ECG-based atrial fibrillation (AF) detection. Subfigures: (**a**) Basic residual convolutional block; (**b**) Full end-to-end model backbone; (**c**) Residual block integrated with self-attention; (**d**) Multi-head self-attention block of the lightweight Transformer encoder.

To achieve accurate and robust AF detection from ECG signals, we proposed RT-AFNet. This architecture synergistically integrated the strengths of 1D-CNNs in extracting local spatiotemporal morphological features with the powerful capability of Transformers in modeling long-range temporal dependencies. The overall structure of the proposed RT-AFNet is shown in Figure 3, which consists of four core modules: a residual connection-based convolutional feature extraction module, a Transformer module for temporal dependency modeling, a multi-scale feature fusion module, and a classification decision module.

#### 2.3.1. Convolutional Feature Extraction Module

The ECG signal is a one-dimensional time series rich in morphological information. The waveform characteristics (shape, width, and intervals) of the P wave, QRS complex, and T wave are critical for distinguishing NSR and AF. Traditional AF features such as R-R interval irregularity often fail to capture subtle morphological changes, including P wave disappearance and baseline fluctuation. 1D-CNNs can effectively extract these spatially correlated morphological features via local receptive fields.

The core of this module is stacked one-dimensional residual convolutional blocks. Compared with traditional deep CNNs, residual connections solve the vanishing gradient problem in deep network training by creating shortcut connections that enable direct information and gradient flow through the network, supporting the training of deeper and more complex architectures. A typical residual block is mathematically expressed as:

(1) $$y=F\left(x,\{W_{i}\}\right)+x,$$

where *x* and *y* are the input and output vectors of the residual block, respectively, and $F\left(x,\{W_{i}\}\right)$ is the residual learning function, typically composed of two or more convolutional layers, batch normalization, and a nonlinear activation function (e.g., ReLU or GeLU). The operation of adding the x forms the shortcut connection.

In this architecture, we designed a feature extraction backbone composed of multiple residual blocks that processes the input raw ECG signal $S\in {\mathbb{R}}^{L\times 1}$ (where *L* denotes the signal length) by first passing it through an initial convolutional layer for preliminary feature mapping and dimensionality enhancement, followed by a series of residual blocks. Each residual block contains two 1D convolutional layers with batch normalization and GeLU activation. This design effectively extracts local morphological features of the ECG signal at different abstraction levels, while ensuring training stability and efficiency via the residual learning mechanism. After processing by this module, the original ECG signal is transformed into a low-dimensional, information-rich feature sequence $F_{\mathrm{cnn}}\in {\mathbb{R}}^{L^{\prime }\times D}$, where $L^{\prime }<L$ denotes the downsampled sequence length and *D* presents the feature dimension.

#### 2.3.2. Transformer Module for Temporal Dependencies Modeling

A core pathophysiological feature of AF is the absolute irregularity of the ventricular rate, which manifests as long-term disordered changes in R-R intervals. Due to their inherent local receptive fields, CNNs struggle to capture these long-range dependencies spanning multiple heartbeats. To address this limitation, we introduced a Transformer Encoder module that computes the dependencies between any two positions in the sequence to build a global contextual representation.

This module processes the feature sequence $F_{\mathrm{cnn}}$ from the convolutional module as input, with positional encoding added to provide the model with sequential order information before feeding into the Transformer encoder. The Transformer encoder comprises *N* identical stacked layers, each containing two core sub-layers: Multi-Head Self-Attention (MHSA) and a Feed-Forward Network (FFN). Residual connections and layer normalization are applied around each sub-layer.

The self-attention mechanism is the core of the Transformer. It projects each element in the input sequence into three spaces: Query (*Q*) Key (*K*) and Value (*V*). The attention weights of each element relative to all other elements are obtained by calculating the similarity between the query and all keys, and the final representation is computed via a weighted sum of the values. The calculation formula is as follows:

(2) $$\mathrm{Attention}(Q,K,V)=\mathrm{softmax}\left(\frac{QK^{T}}{\sqrt{d_{k}}}\right)\cdot V,$$

where *Q*, *K*, and *V* are matrices derived from the input feature sequence $F_{\mathrm{cnn}}$ via independent linear transformations, $d_{k}$ is the dimensionality of the key vector, and the scaling factor $\sqrt{d_{k}}$ prevents excessively large dot product values that would lead to small gradients of the softmax function, thus stabilizing the training process.

The MHSA mechanism enhances the model’s ability to capture information from different perspectives by splitting *Q*, *K*, and *V* into multiple parallel heads and computing attention independently for each head. Each head learns to focus on different feature dependencies in the subspace, enabling the model to capture richer contextual information.

By stacking multiple Transformer encoders, the architecture progressively models complex long-range dependency patterns in ECG feature sequences, such as irregular rhythm changes. This process is particularly critical for distinguishing AF from other arrhythmias with similar local features but regular rhythms (e.g., atrial flutter) [21]. The output of this module, $F_{\mathrm{trans}}\in {\mathbb{R}}^{L^{\prime }\times D}$, is a feature sequence deeply integrated with global temporal context.

#### 2.3.3. Multi-Scale Feature Fusion Module

To further enhance the representational capacity of the architecture, we designed a multi-scale feature fusion module to integrate statistical information from the Transformer output feature map $F_{\mathrm{trans}}$ at different granularities. Diagnostic information in ECG signals may manifest in peak features (e.g., R-wave peaks) and segmental average features (e.g., overall ST-segment morphology). Therefore, we apply global max pooling and global average pooling to the feature sequence in parallel.

Max pooling captures the most prominent features in the feature map, which for ECG signals typically correspond to key diagnostic points such as the peak of the QRS complex [22]. Its operation is expressed as:

(3) $$y_{\max}=\max_{i=1}^{L^{\prime }}\left(F_{\mathrm{trans}}^{\left(i\right)}\right).$$

Average pooling computes the mean of the feature map across the time dimension, preserving overall morphological background information and providing better robustness to noise. Its operation is expressed as:

(4) $$y_{\mathrm{avg}}=\frac{1}{L}\sum_{i=1}^{L^{\prime }}F_{\mathrm{trans}}^{\left(i\right)}.$$

In this architecture, global max pooling and global average pooling are applied across the entire time dimension (length $L^{\prime }$) to generate two D-dimensional feature vectors $F_{\max}$ and $F_{\mathrm{avg}}$. These two pooling strategies provide complementary information, which are concatenated to form a fused feature vector $F_{\mathrm{fused}}$ [23]:

(5) $$F_{\mathrm{fused}}=\mathrm{Concat}\left({F^{\prime }}_{\max},F_{\mathrm{avg}}\right).$$

This fusion method retains all information extracted by both pooling strategies, and allows the subsequent fully connected layer to autonomously learn the optimal weight combination of the features, resulting in a richer and more robust global feature representation compared with a single pooling strategy.

#### 2.3.4. Classification Decision Module

The classification decision module is the final component of the architecture, responsible for mapping the fused high-level feature vector $F_{\mathrm{fused}}$ to the final prediction probability. This module consists of two fully connected layers with a ReLU activation function, and applies the Dropout technique to prevent overfitting:

(6) $$\mathrm{Logits}=W_{2}\left(\mathrm{ReLU}\left(W_{1}F_{\mathrm{fused}}+b_{1}\right)\right)+b_{2},$$

where $W_{1}$, $b_{1}$, $W_{2}$, and $b_{2}$ are learnable weights and biases. Finally, the output Logits are converted into a probability distribution for each category via the softmax activation function, completing the binary classification of the input ECG signal into NSR or AF:

(7) $$P(y=c\;|S)=\frac{e^{z_{c}}}{\sum_{j=1}^{C}e^{z_{j}}},$$

where $Z_{c}$ is the logits value corresponding to class *c*, and *C* is the total number of classes.

In summary, the proposed RT-AFNet effectively extracts local morphological features via the residual CNN module, accurately captures global rhythm dependencies using the Transformer module, and generates a comprehensive feature representation via the multi-scale pooling fusion strategy, ultimately enabling high-precision and high-robustness AF detection.

#### 2.3.5. Training Algorithm

The proposed RT-AFNet is designed for end-to-end ECG signal classification, combining the local feature extraction capability of ResNet with the global sequence modeling advantage of Transformer. The complete training workflow of this hybrid model is detailed in Algorithm 1.

**Algorithm 1.** RT-AFNet Training Pseudocode**Input:** Labeled training dataset $D={\left\{(x_{i},y_{i})\right\}}_{i=1}^{N}$, Validation dataset $D_{val}$, Total training epochs *E*, Initial learning rate η, Fixed Signal length *L* = 4000 (10 s × 400 Hz), Gradient clipping max norm *C* = 1.0, Early stopping patience *P* = 20**Output:** Trained RT-AFNet model $f_{\theta }(\cdot )$ with optimized parameters *θ*1. Randomly initialize model parameters *θ*2. Initialize AdamW optimizer with learning rate η
3. Initialize WCE Loss criterion to mitigate class imbalance4. Initialize SGDR learning rate scheduler for the optimizer5. **for** epoch = 1 **to** *E* **do**6.    Sample mini-batch (x,y) from *D* via weighted random sampler7.    Forward propagation: compute model output logits $\hat{y}=f_{\theta }(x)$
8.    Compute training loss $L=\mathrm{WCE}(\hat{y},y)$
9.    Reset gradients: optimizer.zero_grad()
10.    Backpropagate loss L.backward()
11.    Apply gradient clipping: clip gradients of *θ* to max norm *C*12.    Update model parameters: optimizer.step()
13.    Update learning rate: scheduler.step()
14.    **if** Validation F1-score on $D_{val}$ does not improve for *P* consecutive epochs **then**15.        **break**16.        **end if**17. **end for**

The algorithm processes the following inputs: the labeled dataset $D={\left\{(x_{i},y_{i})\right\}}_{i=1}^{N}$, where $x_{i}$ denotes the input ECG signal segment and $y_{i}$ represents the corresponding ground-truth label for rhythm classification; the validation dataset $D_{val}$ for monitoring model performance; the total training epochs *E* that determines the number of iterative training cycles; the initial learning rate η for optimizer configuration; the fixed signal length *L* = 4000 (corresponding to 10 s × 400 Hz) for standardizing input dimensions; the gradient clipping max norm *C* = 1.0 to prevent gradient explosion; and the early stopping patience *P* = 20 to avoid overfitting. The output of the algorithm is the trained proposed RT-AFNet model $f_{\theta }(\cdot )$ with optimized network parameters *θ*.

Initially, we randomly initialized the network parameters *θ* of the proposed RT-AFNet to provide a stable starting point for subsequent gradient-based optimization. Subsequently, the AdamW optimizer is initialized with the preset learning rate η. This optimizer enables adaptive learning rate adjustment and weight decay regularization during training, effectively mitigating model overfitting. Meanwhile, the Weighted Cross-Entropy (WCE) Loss criterion is initialized as the objective function, specifically designed to address performance degradation caused by class imbalance in ECG rhythm datasets. In addition, the cosine annealing warm restarts (SGDR) scheduler learning rate scheduler is initialized for the optimizer to dynamically adjust the learning rate during training, promoting model convergence and improving generalization performance.

The model underwent iterative optimization over the preset total epochs *E*. In each training epoch, the following operations were executed sequentially. A mini-batch of data was sampled from the labeled dataset *D* via weighted random sampler. This sampling strategy focuses on underrepresented classes, effectively alleviating the impact of class imbalance and improving the model’s ability to learn from minority classes. The preprocessed signal *x* was fed into the proposed RT-AFNet model $f_{\theta }(\cdot )$, and the output classification logits $\hat{y}$ are obtained through forward propagation. These logits represent the model’s preliminary predictions for the input ECG signal category. Finally, the training loss L was calculated using the pre-defined WCE Loss criterion, with the model output logits $\hat{y}$ and the corresponding ground-truth label y as inputs, which follows the formula:

(8) $$L=\frac{1}{B}\sum_{i=1}^{B}L_{WCE}(y_{i},{\hat{y}}_{i}),$$

where *B* is the mini-batch size, $y_{i}$ and ${\hat{y}}_{i}$ are the ground-truth label and model-predicted logits of the *i*-th sample in the mini-batch, respectively. This loss value quantifies the discrepancy between the model’s prediction and the true label, guiding subsequent parameter optimization.

After the loss calculation, backpropagation and parameter update steps were performed. First, optimizer gradients were reset to zero (optimizer.zero_grad()) to prevent gradient accumulation from the previous iteration from affecting the current parameter update. Then, backpropagation was performed on the calculated loss via the L.backward() operation to compute the gradients of all trainable parameters *θ* with respect to the loss function. To prevent gradient explosion during deep hybrid network training, gradient clipping was applied to the model parameters *θ*, limiting the maximum gradient norm to the preset value *C* = 1.0. This operation stabilizes the training process and ensures model convergence. Finally, the AdamW optimizer executed the parameter update step via optimizer.step(), optimizing the network parameters *θ* along the gradient descent direction. After parameter update, the learning rate scheduler was updated via scheduler.step() to adjust the learning rate dynamically.

The WCE Loss adopted in this work was specifically designed to address the class imbalance problem prevalent in ECG classification tasks, such as atrial fibrillation detection and other arrhythmia recognition scenarios. It assigned different weights to different classes, adjusting the loss contribution of each class to mitigate the impact of uneven sample distribution between positive and negative classes, thereby enabling the model to focus more on minority classes during training. The mathematical formula of WCE Loss is defined as follows:

(9) $$L_{WCE}=-\sum_{c=1}^{C}w_{c}\cdot y_{c}\cdot \log({\hat{y}}_{c}),$$

where *C* denotes the total number of ECG rhythm classes, $w_{c}$ represents the weight assigned to the *c*-th class (calculated based on the inverse of class frequency to balance sample distribution), $y_{c}$ is the one-hot encoded ground-truth label for the *c*-th class, and ${\hat{y}}_{c}$ is the model’s predicted probability for the *c*-th class obtained by applying the softmax function to the output logits.

The parameter update process of the model followed the gradient descent optimization rule of the AdamW optimizer, combined with the dynamic learning rate adjustment of the SGDR scheduler. The algorithm optimized the network parameters *θ* by minimizing the WCE loss *L* in each iteration, continuously reducing the discrepancy between the model’s predictions and the true labels. During the training process, if the Validation F1-score on $D_{val}$ did not improve for *P* = 20 consecutive epochs, the training process is terminated early to avoid overfitting. After completing all *E* epochs of iterative training (or early stopping), the algorithm outputs the final optimized RT-AFNet model, which can be applied to subsequent ECG signal classification tasks.

### 2.4. Training Parameters

In our experiments, the training configuration was as follows: The AdamW optimizer was used in combination with the SGDR scheduler. A layer-wise learning rate strategy was employed: the backbone network was assigned a base learning rate of 5 × 10^−4^, the attention modules were set to 1.5× the base rate, and the classifier was set to 2.0× the base rate. The batch size was uniformly set to 64, with a maximum of 150 training epochs. To mitigate overfitting, an early stopping mechanism with a patience of 20 was applied.

### 2.5. Evaluation Metrics

To comprehensively and objectively evaluate the classification performance of the RT-AFNet model for the binary task of Atrial Fibrillation (AF) detection, and to ensure the scientific validity and clinical applicability of the results, this study selected five core evaluation metrics: Accuracy, Precision, Recall, F1-Score, and the Area Under the Receiver Operating Characteristic Curve (AUC). These metrics quantify the model’s performance from different perspectives, including overall classification effectiveness, control of misdiagnosis, control of missed diagnosis, and threshold stability. This aligns with the core requirements for evaluating binary classification tasks in the medical field and comprehensively reflects the practical value of the model in AF detection.

The calculation formulas for the five evaluation metrics are defined below:

#### 2.5.1. Accuracy

Accuracy measures the proportion of correctly classified samples among all samples, reflecting the overall classification performance of the model:

(10) $$\mathrm{Accuracy}=\frac{\mathrm{TP}+\mathrm{TN}}{\mathrm{TP}+\mathrm{TN}+\mathrm{FP}+\mathrm{FN}},$$

where the value ranges from 0 to 1, with a value closer to 1 indicating better overall classification performance.

#### 2.5.2. Precision

Precision focuses on the accuracy of AF-positive predictions, measuring the proportion of true AF samples among all samples predicted as AF. A higher precision corresponds to a lower false positive rate, reducing unnecessary medical interventions and patient psychological burden:

(11) $$\mathrm{Precision}=\frac{\mathrm{TP}}{\mathrm{TP}+\mathrm{FP}},$$

where the value ranges from 0 to 1, with a value closer to 1 indicating higher reliability of AF-positive predictions.

#### 2.5.3. Recall

Recall measures the model’s ability to identify true AF samples, reflecting the proportion of correctly classified AF samples among all true AF samples. A higher recall corresponds to a lower missed diagnosis rate, which is a core clinical requirement for AF screening:

(12) $$\mathrm{Recall}=\frac{\mathrm{TP}}{\mathrm{TP}+\mathrm{FN}},$$

where the value ranges from 0 to 1, with a value closer to 1 indicating a lower risk of missing AF cases.

#### 2.5.4. F1-Score

The F1-Score is the harmonic mean of precision and recall, balancing the model’s ability to control false positives and false negatives. It is particularly suitable for class-imbalanced tasks such as AF detection:

(13) $$F1=2\times \frac{\mathrm{Precision}\times \mathrm{Recall}}{\mathrm{Precision}+\mathrm{Recall}},$$

where the value ranges from 0 to 1, with a value closer to 1 indicating a better balance between precision and recall.

#### 2.5.5. Area Under the Curve (AUC)

AUC is the area under the Receiver Operating Characteristic (ROC) curve, which is plotted with the False Positive Rate (FPR) on the *x*-axis and the True Positive Rate (TPR, i.e., Recall) on the *y*-axis. AUC comprehensively evaluates the model’s discriminative ability across all classification thresholds, independent of a specific decision threshold:

(14) $$\mathrm{AUC}=\int_{0}^{1}\mathrm{TPR}d(\mathrm{FPR}),$$

where FPR is calculated as:

(15) $$\mathrm{FPR}=\frac{\mathrm{FP}}{\mathrm{FP}+\mathrm{TN}},$$

where the AUC value ranges from 0.5 to 1, with a value closer to 1 indicating stronger discriminative power between AF and Non-AF samples.

All of the evaluation metrics mentioned above are calculated based on the confusion matrix for the binary classification task. The core parameters are defined as follows: True Positive (TP) refers to the number of AF samples correctly classified by the model; True Negative (TN) refers to the number of Non-AF samples correctly classified by the model; False Positive (FP) is the number of Non-AF samples incorrectly classified as AF; and False Negative (FN) is the number of AF samples incorrectly classified as Non-AF.

## 3. Results

### 3.1. Experimental Configuration

All experiments were performed on a workstation equipped with an AMD Ryzen 7 7735H processor (base clock 3.20 GHz; Advanced Micro Devices, Inc., Santa Clara, CA, USA) and 16 GB of RAM. The system included an NVIDIA GeForce RTX 4060 Laptop GPU with 8 GB of dedicated video memory. It ran on the Windows 11 Home operating system and utilized an NVMe SSD for storage. The models were implemented using Python 3.8 within the PyCharm 2023 development environment, leveraging deep learning frameworks such as TensorFlow 2.11 and PyTorch 2.4.1, together with common libraries including NumPy 1.24.4, scikit-learn 1.3.2, and Transformers 4.46.3. This hardware and software configuration ensured reproducible and efficient experimental runs.

### 3.2. Quantitative Classification Performance

To comprehensively validate the AF detection performance and generalization capability of our proposed RT-AFNet architecture, we performed systematic verification experiments on three widely used public datasets, namely CPSC2018, CinC2017 and MIT-BIH AF. The five core evaluation metrics were used to quantify the model’s performance for the binary AF vs. Non-AF classification task. The performance results on the three datasets are presented in Table 2.

On the CPSC2018 and MIT-BIH AF datasets, both of which use standard Lead II, the model achieved F1 scores of 99.76% and 96.20%, respectively. On the CinC2017 dataset, which employs a Lead I equivalent via the AliveCor Kardia device, the model achieved an F1 score of 97.47%. Despite the difference in lead orientation between CinC2017 (Lead I) and the other two datasets (Lead II), the model maintained consistently strong performance across all three without any lead-specific fine-tuning. This cross-lead robustness suggests that RT-AFNet captures lead-invariant features for AF detection: the ResNet backbone extracts local morphological patterns that retain diagnostic waveform characteristics across lead configurations, while the Transformer module models global rhythm irregularity, a temporal property independent of lead orientation. This inherent property is a practical advantage for deployment across heterogeneous wearable ECG devices.

### 3.3. Confusion Matrix Analysis

**Figure 4:**
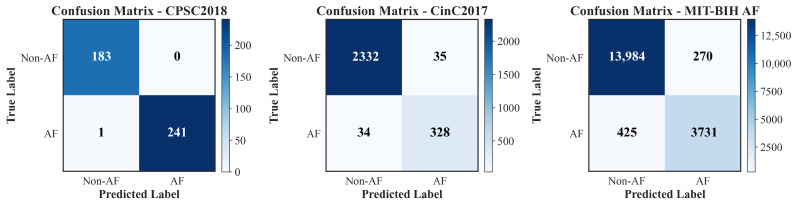
Confusion matrices of the proposed RT-AFNet model on the CPSC2018, CinC2017, and MIT-BIH AF test sets. The horizontal axis represents the predicted label, and the vertical axis represents the true label.

To intuitively illustrate the classification performance and misclassification distribution of the model on each dataset, we plotted the confusion matrices for the test sets of CPSC2018, CinC2017, and MIT-BIH AF datasets, as shown in Figure 4. The matrices quantify the model’s binary classification results for AF and Non-AF categories in terms of true negatives (TN), false positives (FP), false negatives (FN), and true positives (TP). On the CPSC2018 dataset, the model correctly identified 183 Non-AF samples and 241 AF samples, with only 0 FP and 1 FN, reflecting an extremely low misclassification rate for both categories. For the larger-scale CinC2017 dataset consisting of short-term single-lead ECG recordings, the model achieved 2332 TN, 328 TP, 35 FP, and 34 FN, maintaining high classification accuracy in ambulatory ECG monitoring scenarios. On the long-term MIT-BIH AF dataset, a widely recognized challenging benchmark with abundant paroxysmal AF episodes, the model correctly classified 13,984 Non-AF samples and 3731 AF samples, with only 270 FP and 425 FN, demonstrating excellent capability to capture transient AF episodes while minimizing false alarms in long-term continuous monitoring. Across all three datasets, the model consistently exhibited a high true positive rate and true negative rate, with limited false positive and false negative events, verifying its stable and reliable classification performance for AF detection.

### 3.4. ROC Curve and AUC Evaluation

**Figure 5:**
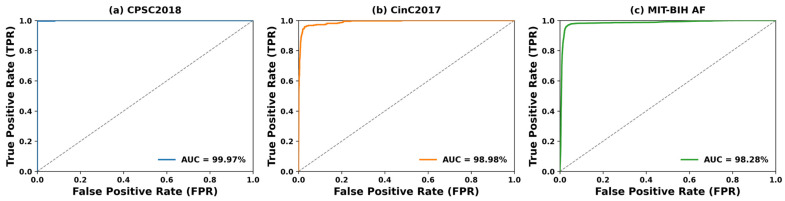
ROC curves of the proposed RT-AFNet on the test sets: (**a**) CPSC2018 dataset; (**b**) CinC2017 dataset; (**c**) MIT-BIH AF dataset. The gray dashed line represents the baseline of random classification with an AUC of 0.5.

The receiver operating characteristic (ROC) curves of the proposed model on the test sets of the three datasets are presented in Figure 5, which further characterizes the model’s overall discriminative ability across different classification thresholds. The ROC curve plots the true positive rate (TPR, sensitivity) against the false positive rate (FPR, 1-specificity) at various decision thresholds, and the area under the ROC curve (AUC) serves as a comprehensive metric for the model’s global classification performance. As shown in Figure 5, the ROC curves of the model on all three datasets are tightly close to the upper-left corner of the coordinate system, which represents the ideal classification performance with 100% TPR and 0% FPR. The model achieved AUC values of 99.97%, 98.98%, and 98.28% on the CPSC2018, CinC2017, and MIT-BIH AF datasets, respectively, all approaching the perfect value of 1.0. These results further confirm that the proposed model has excellent discriminative power for distinguishing AF from Non-AF ECG signals.

### 3.5. SHAP-Based Model Interpretability

Despite the superior classification performance achieved by deep learning models, they are often criticized as “black boxes” due to their multi-layer non-linear structure, which makes the model’s decision-making process difficult for humans to understand. In clinical practice, the interpretability of a diagnostic model is as critical as its diagnostic accuracy, as it can help clinicians understand the basis of the model’s decisions, enhance trust in the algorithm, and provide auxiliary evidence for clinical decision-making. To address this issue, we used the SHapley Additive exPlanations (SHAP) method, a game-theoretic approach that provides a unified framework for interpreting the predictions of any machine learning model. Specifically, SHAP assigns a unique Shapley value to each input feature, which quantifies the additive contribution of that feature to the model’s final prediction. A positive Shapley value indicates that the feature has a positive contribution to the model’s prediction of AF, while a negative value indicates a negative contribution towards AF classification (i.e., supporting the prediction of Non-AF). In this study, we used the gradient explainer to calculate the Shapley values for each time step of the input ECG signals, enabling us to interpret the model’s decision-making process at the patient level.

**Figure 6:**
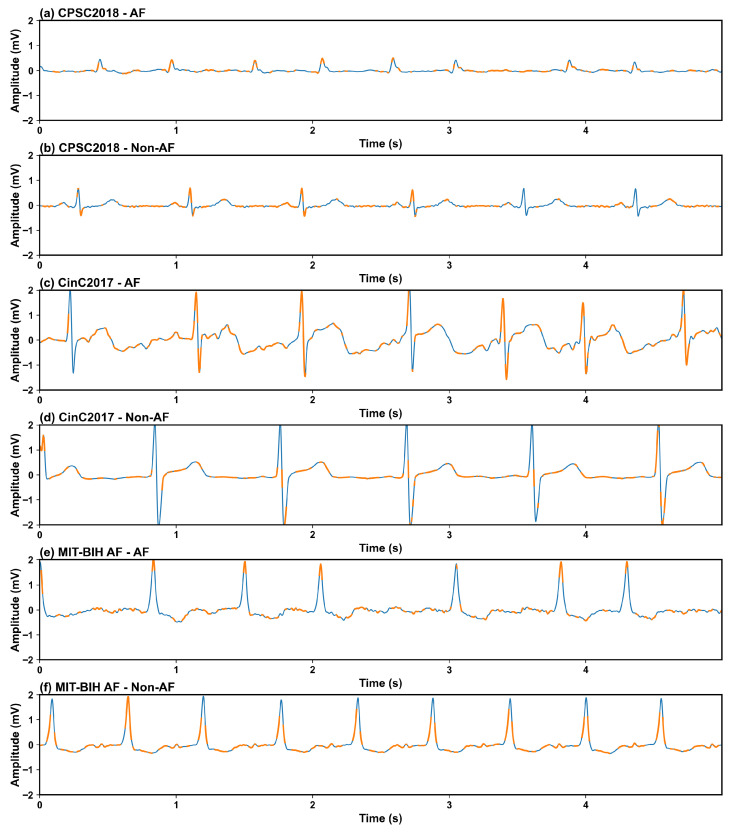
Patient-level SHAP interpretation of the model’s prediction results for representative ECG samples. The raw ECG signals are plotted in blue, and the regions with high positive Shapley values (key contributors to the model’s classification decision) are highlighted in orange: (**a**) AF sample from CPSC2018; (**b**) Non-AF sample from CPSC2018; (**c**) AF sample from CinC2017; (**d**) Non-AF sample from CinC2017; (**e**) AF sample from MIT-BIH AF; (**f**) Non-AF sample from MIT-BIH AF.

The patient-level interpretation results of the model on representative AF and Non-AF samples from the three datasets are visualized in Figure 6. For the AF samples from all three datasets (Figure 6a,c,e), the high-contribution regions highlighted by Shapley values were mainly concentrated in the areas corresponding to the absence of P waves and irregular RR intervals, which are the gold-standard diagnostic criteria for AF in clinical practice. In contrast, for the Non-AF (normal sinus rhythm) samples (Figure 6b,d,f), the regions with high Shapley values were aligned with regular P waves, stable PR intervals, and normal QRS complexes, which are the typical characteristics of normal sinus rhythm. These observations demonstrate that the decision-making basis of the proposed model is highly consistent with clinical AF diagnostic rules, rather than relying on spurious correlations or noise in the ECG data. This patient-level interpretability not only verifies the rationality of the model’s feature learning, but also provides intuitive visual evidence for clinicians to verify the model’s diagnostic results.

### 3.6. t-SNE Feature Visualization

t-SNE is a widely adopted non-linear dimensionality reduction technique that preserves both the local and global structural characteristics of high-dimensional feature data in the low-dimensional embedding space. This enables intuitive visualization of the inter-class separability and intra-class compactness of the features learned by deep models.

**Figure 7:**
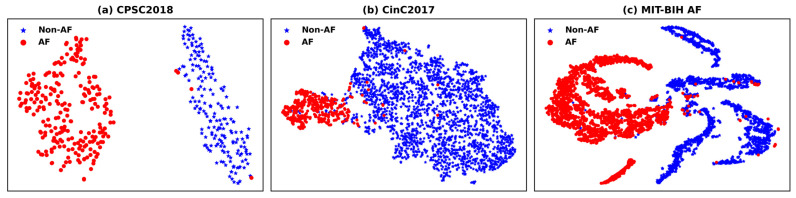
Two-dimensional t-SNE visualization of the high-level features extracted by the proposed model from the test set samples: (**a**) CPSC2018 dataset; (**b**) CinC2017 dataset; (**c**) MIT-BIH AF dataset. Red dots represent AF samples, and blue stars represent Non-AF samples.

To further validate the effectiveness and discriminability of the high-level features learned by the proposed RT-AFNet model, we employed t-SNE to reduce the dimensionality of the deep features extracted from the test set samples of each dataset. The resulting distribution of AF and Non-AF samples in the two-dimensional feature space is visualized in Figure 7.

For the CPSC2018 dataset shown in Figure 7a, AF and Non-AF samples form two completely independent, well-separated clusters with negligible overlap in the 2D embedding space, with only a tiny number of outlier samples crossing the inter-class boundary. It is worth noting that the CPSC2018 dataset has the smallest sample size among the three datasets. Since the duration of each original ECG recording in this dataset is relatively short, only one or two valid analysis windows can be segmented from each recording, resulting in a test set with only 425 samples (AF = 242, Non-AF = 183). Even with such a limited sample scale, the model still achieves clear class separation in the feature embedding space. This demonstrates that the proposed model can learn highly discriminative and robust high-dimensional features even in small-sample scenarios, fully reflecting the excellent feature extraction capability of our hybrid architecture.

In contrast, for the CinC2017 dataset shown in Figure 7b, although AF and Non-AF samples form two relatively distinct main clusters in the embedding space, there is a non-negligible degree of overlap between the two categories. A small number of samples from each class are distributed within the cluster of the opposite category. Despite this partial overlap in the low-dimensional projection, the proposed model still achieved excellent and stable classification performance on this dataset. This phenomenon indicates that the model has learned complex non-linear decision boundaries in the original high-dimensional feature space, which can accurately distinguish AF and Non-AF samples even when their low-dimensional embeddings are not fully separated. These results further verify the strong non-linear feature modeling capability of our RT-AFNet framework.

For the MIT-BIH AF dataset shown in Figure 7c, a widely recognized challenging benchmark for AF detection with abundant long-term paroxysmal AF episodes, AF and Non-AF samples are almost completely divided into two independent, highly separated clusters in the 2D embedding space. Only an extremely small number of outlier samples cross the category boundary. This nearly perfect inter-class separation in the low-dimensional space directly demonstrates that the high-level features learned by the proposed model possess exceptional inter-class discriminability, even for the complex, intermittent, and variable paroxysmal AF signals contained in this long-term ECG recording dataset.

### 3.7. Ablation Study

To quantify the contribution of each architectural component and validate the design choices of RT-AFNet, we conducted an ablation study on the CinC2017 dataset. Four model variants were evaluated: ResNet-only, Transformer-only, without multi-scale fusion (retaining only average pooling), and without weighted cross-entropy loss (using standard cross-entropy). Table 3 summarizes the results.

The ResNet-only configuration, which relies primarily on local morphological features, achieved an F1-score of 95.04%, compared to 97.47% for the full model. The Transformer-only configuration, which models global rhythm patterns without detailed waveform analysis, achieved 92.72%. These results demonstrate two points. First, each branch alone provides substantial discriminative power, confirming that both morphological and temporal features carry diagnostic information for AF detection. Second, the combined architecture outperforms either single-branch configuration by 2.43 and 4.75 percentage points in F1-score, respectively, directly validating that morphological analysis and temporal irregularity modeling are complementary and that their synergistic integration constitutes the core advantage of RT-AFNet over methods relying predominantly on a single feature type.

Removing the multi-scale fusion module decreased the AUC by 0.60 percentage points, and replacing the weighted cross-entropy loss with standard cross-entropy reduced it by 0.13 percentage points. Although the full model already achieves strong performance, every ablation variant showed a consistent decrease across all metrics, confirming that each component contributes to the final result. Together, the ablation results validate that all components of RT-AFNet are necessary for its optimal performance, with the integration of ResNet-based morphological feature extraction and Transformer-based temporal dependency modeling serving as the primary performance driver.

### 3.8. Cross-Database Generalization

To rigorously assess the generalization capability of RT-AFNet across different acquisition systems and patient populations without any dataset-specific retraining, we conducted zero-shot cross-database evaluations. A model trained on one dataset was directly evaluated on the test set of another dataset. Table 4 summarizes the results for three transfer scenarios forming a complete evaluation cycle across all three datasets.

When trained on CinC2017 (short single-lead recordings from a consumer device) and tested on MIT-BIH AF (long-term clinical recordings), RT-AFNet achieved an F1 score of 95.80%, demonstrating that features learned from consumer-grade short recordings transfer effectively to clinical-grade long-term data. In the reverse direction, MIT-BIH AF to CPSC2018 yielded an F1 score of 94.35%, confirming robust generalization from long-term clinical data to multi-lead clinical recordings. The CPSC2018 to CinC2017 transfer, the most challenging due to limited CPSC2018 training samples, still achieved an F1 score of 84.76%. These results confirm that RT-AFNet learns genuine, dataset-invariant features of AF rather than overfitting to domain-specific artifacts.

### 3.9. Error Analysis on Confounding Rhythms

To investigate whether classification errors are associated with specific ECG patterns, we conducted a focused error analysis on the MIT-BIH AF test set. Using the original rhythm annotations, we reconstructed fine-grained labels for all test windows, separating atrial flutter (AFL) from the binary AF/Non-AF labels used in training. AV junctional rhythm (J) segments were absent from the test set, as the limited number of J segments in the MIT-BIH AF database (only 32 windows across all records) were all allocated to the training or validation sets under patient-based stratified splitting. The classification results across rhythm types are summarized in Table 5.

**Figure 8:**
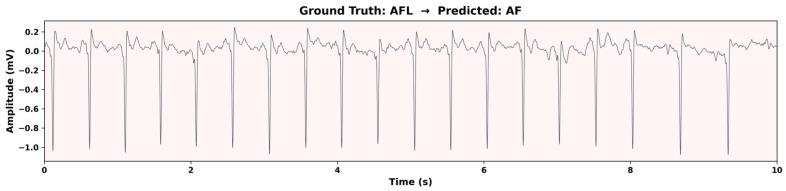
Representative misclassification case: an AFL segment misclassified as AF.

Among AFL segments, 84.07% were misclassified as AF, confirming that atrial flutter with variable AV block represents a significant challenge for binary AF classifiers. Figure 8 illustrates a representative AFL segment misclassified as AF. The waveform exhibits characteristic saw-tooth flutter waves and irregular ventricular response that closely mimic the disorganized atrial activity of AF, highlighting why even a model combining morphological and temporal analysis can confuse these two rhythms in the absence of explicit AFL training. In contrast, normal sinus rhythm segments were classified with high accuracy (1.89% false AF rate), demonstrating the model’s strong specificity on the most prevalent Non-AF class. These results directly confirm that classification errors are strongly associated with specific ECG patterns, particularly AFL, which is inherently difficult to distinguish from AF in single-lead recordings when AV conduction is variable.

### 3.10. Complexity Analysis

To evaluate the deployment feasibility of the proposed RT-AFNet in portable and wearable ECG monitoring devices, we conducted a comprehensive computational complexity and inference efficiency analysis.

Table 6 summarizes the architectural specifications of RT-AFNet, measured on a single NVIDIA GeForce RTX 4060 Laptop GPU. The model contains 17.02 M trainable parameters and requires 3.26 GFLOPs for a single 10-s ECG segment (4000 samples at 400 Hz). The model weights occupy 68.27 MB of storage, and the peak GPU memory consumption during inference is 82.40 MB.

**Figure 9:**
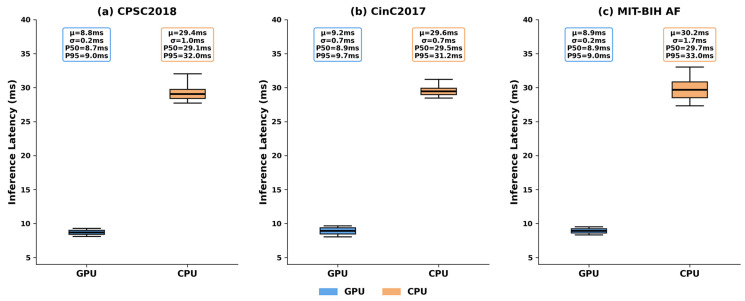
Single-sample inference latency distribution of the proposed RT-AFNet on GPU and CPU platforms. μ: mean latency; σ: standard deviation of latency; P50: 50th percentile latency (median); P95: 95th percentile latency. (**a**) CPSC2018 dataset. (**b**) CinC2017 dataset. (**c**) MIT-BIH AF dataset.

Table 7 reports the single-sample inference latency and batch throughput on both GPU and CPU across all three test datasets. The latency distribution is visualized in Figure 9. The mean GPU latency ranges from 8.76 ms (CPSC2018) to 9.19 ms (CinC2017), corresponding to a real-time factor of over 800× for 10-s ECG segments. On CPU, the mean latency ranges from 29.39 ms to 30.21 ms, still well below the segment duration and suitable for edge devices without GPU acceleration. The GPU throughput exceeds 1000 samples per second on all datasets, confirming that RT-AFNet can process continuous streaming ECG data in real time.

The overall forward propagation complexity of the proposed model is O(n), where n is the input signal length (n = 4000 for 10-s segments at 400 Hz). The complexity breakdown of each core module is summarized in Table 8.

Unlike pure Transformer architectures, which have a native O(n^2^) computational complexity that grows quadratically with input signal length, the self-attention module in our hybrid architecture takes as input the feature sequence downsampled by the ResNet backbone. This results in a fixed and greatly reduced sequence length L, making the computational overhead of the self-attention module a constant value independent of the original input signal length n. This design completely avoids the excessive computational complexity and poor scalability of pure Transformer models when processing long-term ECG signals. Both the theoretical analysis and the empirical measurements confirm that the proposed model is inherently lightweight and scalable, providing a solid foundation for deployment on resource-constrained edge devices.

In summary, the experimental results in this section demonstrated that the proposed RT-AFNet model achieved excellent and stable classification performance across three diverse public AF datasets. The confusion matrices confirmed its low misclassification rates, and the ROC curves validated its strong discriminative ability across all decision thresholds. The ablation study verified that each architectural component contributes to the overall performance, with the integration of ResNet-based morphological feature extraction and Transformer-based temporal dependency modeling serving as the primary performance driver. The cross-database evaluations confirmed that the model learns genuine, dataset-invariant features of AF rather than overfitting to domain-specific artifacts. The error analysis on confounding rhythms revealed that classification errors are strongly associated with specific ECG patterns, particularly atrial flutter, which remains inherently difficult to distinguish from AF in single-lead recordings. The SHAP analysis demonstrated that the model’s decision-making process aligns with clinical AF diagnostic criteria, and the t-SNE visualization verified the excellent inter-class separability of its learned high-level features. Moreover, the complexity analysis confirmed that the model is inherently lightweight and scalable, with linear forward propagation complexity O(n), effectively overcoming the quadratic complexity limitation of pure Transformer architectures. Taken together, these results establish the proposed RT-AFNet as a robust, interpretable, and computationally efficient deep learning solution for automated atrial fibrillation screening, well-suited for deployment in portable and wearable ECG monitoring devices.

## 4. Discussion

The proposed RT-AFNet achieves excellent and stable detection performance across three public AF datasets with distinct data characteristics. To further validate the superiority of our model, this section conducted an in-depth performance comparison between RT-AFNet and existing state-of-the-art AF detection methods, with the complete comparison results summarized in Table 9.

### 4.1. Comparison on the CPSC2018 Dataset

The CPSC2018 dataset contains a large number of multi-lead ECG recordings from real clinical settings, with a complex data distribution that demands strong anti-interference and generalization capabilities. Several deep learning methods have been evaluated on this dataset. MTNN [24] employed a multi-task learning framework and reported an AUC of 97.70% on this dataset, but its recall was only 80.80%, indicating difficulty in identifying low-amplitude or short-duration AF signals. lightX3ECG [25], a lightweight and explainable deep learning system designed for 3-lead ECGs, achieved a recall of 78.62% and an F1 score of 80.04% on the CPSC2018 dataset. STFAC-ECGNet [26] utilized a spatio-temporal fusion network and obtained a recall of 75.60% and an AUC of 93.30%. These methods show that while AUC can be high, recall remains a challenge. In contrast, our proposed RT-AFNet, which integrated ResNet and Transformer with multi-scale feature fusion, achieved a recall of 99.76%, an AUC of 99.97%, and a precision of 99.77% on the same dataset, demonstrating its ability to capture both local and global features effectively.

### 4.2. Comparison on the CinC2017 Dataset

The CinC2017 dataset consists of short-duration, single-lead ECG recordings, simulating the data acquisition scenario of wearable devices. This places high demands on feature compression and efficient recognition. VGGNet [27] uses a dual-channel neural network fed with time-frequency spectrograms and Poincaré plots; it reported an overall F1 score of 83.00% and an accuracy of 87.00% on this dataset. TCN-ResNet [28] combined temporal convolutional networks with residual networks, achieving an accuracy of 97.00%, a recall of 92.00%, and an F1 score of 87.00%, but with a noticeable imbalance between recall and precision. CTRhythm [29] integrated CNN with a Transformer encoder and obtained an accuracy of 85.40% and an overall F1 score of 83.10%. These methods highlight the trade-offs between different metrics. Our proposed RT-AFNet, achieved highly balanced classification performance, with accuracy, recall, and F1-score all reaching 97.47%. This demonstrates the model’s strong robustness to the inherent variability and noise in single-lead wearable ECG recordings.

### 4.3. Comparison on the MIT-BIH AF Dataset

The MIT-BIH AF dataset is a widely used benchmark containing long-term recordings with paroxysmal AF episodes, requiring the model to capture rhythm irregularities accurately. ResNet [30] was applied to this dataset and achieved a high recall of 98.08% but a low accuracy of 71.32%, suggesting severe overfitting and poor discrimination between AF and other rhythm abnormalities. MGNN [31] employed a multiscale grouped convolutional network and obtained a precision of 96.16% but a recall of only 79.99%, leading to a high risk of missed AF diagnoses. CNN + LSTM [32] combined convolutional and recurrent layers to achieve a more balanced performance with a recall of 91.81%, accuracy of 92.48%, and F1 score of 93.28%. Our RT-AFNet, which leveraged both local feature extraction and long-range dependency modeling, achieved a recall of 96.22%, accuracy of 96.22%, and F1 score of 96.20% on this dataset. These results demonstrate the model’s capability to accurately detect AF episodes while minimizing false positives.

### 4.4. Expected Model Behavior on Confounding Arrhythmias and Limitations

Although this study focused on binary AF versus Non-AF classification, understanding model behavior in the presence of other arrhythmias is critical for evaluating real-world screening performance. We provide a qualitative analysis of expected model behavior based on the architecture of RT-AFNet.

Premature contractions (PAC/PVC). Premature beats induce momentary RR interval irregularity, a well-known cause of false positives for AF detectors relying solely on rhythm irregularity [33,34]. In RT-AFNet, the ResNet backbone first extracts local QRS morphology: PVCs present a characteristically wide and bizarre QRS complex, while PACs typically show a normal QRS morphology preceded by an ectopic P wave. The Transformer module then processes these ResNet-derived features to model temporal dependencies across the entire segment, and can recognize that the rhythm irregularity induced by a premature contraction is non-sustained and punctate, contrasting with the continuously chaotic pattern of AF. Because the ResNet provides beat-level morphological features that already encode diagnostically relevant distinctions between normal and ectopic beats, the Transformer has a principled basis for distinguishing isolated irregularity from sustained AF. We therefore expect RT-AFNet to exhibit strong resilience against premature contractions.

Conduction abnormalities (e.g., bundle branch blocks). LBBB and RBBB are characterized by widened QRS complexes with consistent, regular RR intervals. The ResNet backbone extracts the widened QRS morphology, while the Transformer models the temporal regularity of ventricular intervals across the entire segment. Since the rhythm remains regular, the Transformer’s global temporal context can guide the classification toward Non-AF even when the ResNet encounters unfamiliar QRS widths [35].

Atrial flutter (AFL) and junctional rhythms (J). These represent the most significant challenges and are widely recognized as common failure modes for both RR-based and morphology-based AF algorithms. Our error analysis on the MIT-BIH AF database (Section 3.8) confirms a high false positive rate on AFL segments, with 84.07% (306/364) of AFL windows misclassified as AF, indicating that organized flutter waves and disorganized fibrillatory waves remain difficult to differentiate in single-lead recordings, even with combined morphological and temporal analysis. This is consistent with prior findings that distinguishing AF from AFL remains a challenge even for AI-based algorithms [36,37]. AV junctional rhythm segments were absent from the test set, as all J segments in the database were allocated to the training or validation sets under patient-based splitting. In principle, the Transformer’s sensitivity to overall rhythm regularity, which is typically preserved in junctional escape rhythms, may help distinguish J from AF.

### 4.5. Effect of Data Augmentation Strategy

To investigate the impact of selective data augmentation, we compared three training configurations on the CinC2017 dataset: no augmentation, augmentation of AF samples only, and augmentation of both AF and Non-AF samples. Table 10 summarizes the results.

Without any augmentation, the model achieved an F1-score of 80.45% and an AUC of 57.07%, confirming that class imbalance substantially degrades detection performance and that augmentation is necessary. When both AF and Non-AF samples were augmented, the model collapsed (F1 = 3.11%, AUC = 51.95%). We attribute this to the signal-level effects of random masking and Gaussian noise on Non-AF rhythms. Normal sinus rhythm is defined by regular P-QRS-T morphology and stable R-R intervals. Applying random masking can obscure the P wave, and Gaussian noise can introduce amplitude perturbations. Both effects may cause augmented Non-AF segments to superficially resemble the irregular, low-amplitude, P-wave-absent appearance of AF, creating a distribution shift between the augmented training data and the clean test data that destroyed discriminative ability. The current strategy of augmenting only the underrepresented AF class achieved an F1-score of 97.47% and an AUC of 98.98% with well-balanced precision and recall. By addressing class imbalance without corrupting the feature distribution of the majority class, this selective augmentation provides the optimal training configuration.

In summary, the proposed RT-AFNet demonstrates strong and balanced performance across three major public AF datasets. This performance is driven by the synergistic interplay of ResNet-based local morphological feature extraction and Transformer-based global temporal dependency modeling within a multi-scale feature fusion framework. The qualitative analysis of confounding arrhythmias indicates expected resilience against premature contractions and regular-rhythm conduction abnormalities, while atrial flutter remains inherently challenging in single-lead recordings. The selective augmentation of the underrepresented AF class proved essential for addressing class imbalance without corrupting the feature distribution of the majority class. The current model is best deployed as a high-sensitivity screening tool, with multi-class extension using comprehensive datasets constituting a key direction for future work.

## 5. Conclusions

In this study, we proposed RT-AFNet, a novel spatiotemporal hybrid architecture integrating a ResNet backbone, a lightweight Transformer, and multi-scale feature fusion for the robust automated detection of atrial fibrillation (AF). Extensive experiments across three public datasets (CPSC2018, CinC2017, and MIT-BIH AF) demonstrated that RT-AFNet achieved F1-scores of 99.76%, 97.47%, and 96.20%, with corresponding AUC values of 99.97%, 98.98%, and 98.28%, respectively, delivering stable and state-of-the-art performance across all benchmarks. Future work will focus on three directions: extending RT-AFNet to multi-lead inputs for more comprehensive diagnostic information, expanding the framework to multi-class classification for comprehensive arrhythmia differentiation, and developing advanced lightweighting strategies, such as model quantization and network pruning, to further optimize its parameter footprint for ubiquitous deployment on ultra-low-power edge microcontrollers.

## Figures and Tables

**Table 1:** Dataset partition and sample statistics after preprocessing.

Dataset	Recordings			Samples					
	Train	Val	Test	Train	Val	Test	AF	Non-AF	Total
CPSC2018	1496	321	322	3063	415	425	2702	1201	3903
Cinc2017	4082	876	876	14,494	2711	2729	4083	15,851	19,934
MIT-BIH AF	14	4	5	77,016	14,728	18,410	60,033	50,121	110,154

**Table 2:** Classification performance of the proposed RT-AFNet model on three test datasets.

Dataset	Recall (%)	AUC (%)	Accuracy (%)	Precision (%)	F1-Score (%)
CPSC2018	99.76	99.97	99.76	99.77	99.76
CinC2017	97.47	98.98	97.47	97.47	97.47
MIT-BIH AF	96.22	98.28	96.22	96.19	96.20

**Table 3:** Ablation study results on the CinC2017 dataset.

Configuration	Accuracy (%)	Precision (%)	Recall (%)	F1-Score (%)	AUC (%)
ResNet-only	94.98	95.12	94.98	95.04	97.13
Transformer-only	92.67	92.78	92.67	92.72	94.66
w/o Multi-Scale Fusion	97.25	97.28	97.25	97.27	98.38
w/o Weighted Loss	97.36	97.39	97.36	97.37	98.85
RT-AFNet	97.47	97.47	97.47	97.47	98.98

**Table 4:** Cross-database generalization performance of RT-AFNet.

Source	Target	Precision (%)	Recall (%)	F1-Score (%)
CinC2017	MIT-BIH AF	96.10	95.71	95.80
MIT-BIH AF	CPSC2018	94.35	94.35	94.35
CPSC2018	CinC2017	85.19	84.39	84.76

**Table 5:** Fine-grained error analysis on the MIT-BIH AF test set.

Rhythm	Total Windows	Predicted AF	Predicted Non-AF	Error Rate
AFL	364	306	58	84.07%
N	14,254	270	13,984	1.89%
AF	3792	3425	367	9.68%

**Table 6:** Model architecture specifications of RT-AFNet.

Metric	Value
Trainable Parameters (M)	17.02
FLOPs (G)	3.26
Model Size (MB)	68.27
GPU Inference Memory (MB)	82.40

**Table 7:** Inference latency and throughput of RT-AFNet across three datasets.

Dataset	Device	Mean Latency (ms)	Standard Deviation (ms)	P50 (ms)	P95 (ms)	Throughput (sps)
CPSC2018	GPU	8.76	0.18	8.71	8.97	1010.47
	CPU	29.39	1.00	29.08	32.04	56.93
CinC2017	GPU	9.19	0.69	8.93	9.67	1042.56
	CPU	29.64	0.71	29.46	31.22	57.54
MIT-BIH AF	GPU	8.95	0.17	8.93	9.03	1045.16
	CPU	30.21	1.74	29.70	33.04	56.98

**Table 8:** Theoretical computational complexity of core modules in RT-AFNet.

Module	Computational Complexity	Note
ResNet Backbone	O(n)	Linear with the input signal length n, dominated by 1D convolutional layers with fixed kernel sizes
Lightweight Self-Attention Block	O(L^2^·d)	L is the fixed sequence length (250/500) after downsampling by the ResNet backbone, which is a constant independent of the original input length n; d is the feature dimension
Global Adaptive Average Pooling + Max Pooling	O(1)	Global pooling operation, independent of the input signal length
Fully Connected Classifier	O(1)	Fixed feature dimensions (1024→256→2), independent of the input signal length
Overall Forward Pass	O(n)	Dominated by the ResNet backbone, with no O(n^2^) complexity bottleneck

**Table 9:** Performance comparison on three datasets between our model and cutting-edge methods.

Dataset	Model	Recall (%)	AUC (%)	Accuracy (%)	Precision (%)	F1-Score (%)
CPSC2018	MTNN [24]	80.80	97.70	96.60	85.20	82.70
	lightX3ECG [25]	78.62	—	96.28	82.09	80.04
	STFAC-ECGNet [26]	75.60	93.30	89.40	77.80	76.70
	The proposed RT-AFNet	99.76	99.97	96.76	99.77	99.76
CinC2017	VGGNet [27]	82.00	—	87.00	—	83.00
	TCN-ResNet [28]	92.00	—	97.00	92.00	87.00
	CTRhythm [29]	—	—	85.40	—	83.10
	The proposed RT-AFNet	97.47	98.98	97.47	97.47	97.47
MIT-BIH AF	ResNet [30]	98.08	—	71.32	68.22	80.45
	MGNN [31]	79.99	—	87.42	96.16	87.19
	CNN + LSTM [32]	91.81	—	92.48	94.86	93.28
	The proposed RT-AFNet	96.22	98.28	96.22	96.19	96.20

**Table 10:** Effect of data augmentation strategy on CinC2017.

Augmentation Strategy	Accuracy (%)	Precision (%)	Recall (%)	F1-Score (%)	AUC (%)
No augmentation	86.48	75.20	86.48	80.45	57.07
AF only	97.47	97.47	97.47	97.47	98.98
Both AF and Non-AF	13.26	1.76	13.26	3.11	51.95

## Data Availability

The datasets used in this study are all publicly available from the PhysioNet repository. The CPSC2018 database is part of the PhysioNet/Computing in Cardiology Challenge 2020. It is available at: (https://physionet.org/content/challenge-2020/1.0.2/training/cpsc_2018/#files-panel, accessed on 30 November 2025), The CinC2017 dataset is available at: (https://physionet.org/content/challenge-2017/get-zip/1.0.0/, accessed on 30 November 2025), The MIT-BIH Atrial Fibrillation Database is available at: (https://www.physionet.org/content/afdb/1.0.0/, accessed on 30 November 2025). All data were used in accordance with the original licenses provided by the source.
